# When muscle quivers and undulates

**DOI:** 10.1002/ccr3.6086

**Published:** 2022-07-22

**Authors:** Alex Rebello, Mohd Asif, Kumbha Dhanusha, Bandi Haritha, Nangadda Narmada, Ram Chandra Poudel

**Affiliations:** ^1^ Department of Neurology All India Institute of Medical Sciences Mangalagiri India; ^2^ Department of General Medicine All India Institute of Medical Sciences Mangalagiri India; ^3^ Department of General Medicine Nova Hospital Dhangadhi Nepal

**Keywords:** acquired neuromyotonia, CASPR2 antibodies, Isaacs syndrome, Myokymia

## Abstract

We describe a patient who presented with fatigue and pulling sensation in his lower limbs. He had continuous muscle contractions over his trunk (myokymia) which pointed towards the diagnosis of Isaacs syndrome which was confirmed by strongly positive CASPR2 antibodies in blood.

## Abstract 2

Isaacs syndrome is a neuromuscular disorder with progressive stiffness and continuous contractions in muscles. The cause is usually uncertain; however, many are autoimmune mediated. The diagnosis may be made clinically by a proper examination, which can reveal myokymia. The presence of antibodies against voltage‐gated potassium channels confirms the diagnosis.

A 40‐year‐old man presented with history of fatigue, low back ache, and dragging sensation in both his lower limbs since 1 month. On examination, there was continuous undulatory twitching of muscles (myokymia) over his right upper back (Video S1). He had normal muscle power and brisk deep tendon reflexes. MRI spine did not show any significant abnormality except early cervical spondylotic changes. He was clinically suspected to have Isaacs syndrome. CASPR2 antibodies in blood were strongly positive and confirmed the diagnosis.^1^ Workup for systemic autoimmune diseases and malignancies was unremarkable. He was given pulse of high dose intravenous methylprednisolone 1 g for 5 days and oral phenytoin at a dose of 5 mg/kg daily. His symptoms were significantly reduced, and myokymia disappeared on follow‐up after 1 month.

## AUTHOR CONTRIBUTIONS

Alex Rebello conceptualized, organized, and executed the study, prepared the manuscript, collected the data, and involved in review. Mohd. Asif, Bandi Haritha, Nangadda Narmada, and Kumbha Dhanusha prepared the manuscript and collected the data. Ram Chandra Poudel executed the study, and involved in review and critique.

## CONFLICT OF INTEREST

None.

## CONSENT

Written informed consent was obtained from the patient to publish this report in accordance with the journal's patient consent policy.

## Supporting information


Video S1

## Data Availability

The data that support the findings of this study are available from the corresponding author upon reasonable request.
